# Nanocrystallization Effectively Improves the Oral Efficacy of an Antileishmanial Chalcone

**DOI:** 10.3390/pharmaceutics17040399

**Published:** 2025-03-21

**Authors:** Maria Paula Gonçalves Borsodi, Wallace Pacienza-Lima, Jaqueline Correia Villaça Menezes, Douglas Escrivani-Oliveira, Natalia Arruda-Costa, Alcides José Monteiro da Silva, Lucio Mendes Cabral, Patrick G. Steel, Ariane de Jesus Sousa-Batista, Bartira Rossi-Bergmann

**Affiliations:** 1 Institute of Biophysics Carlos Chagas Filho, Federal University of Rio de Janeiro, Rio de Janeiro 21941-902, Brazil; mariapaula@biof.ufrj.br (M.P.G.B.); wallace.pacienza@gmail.com (W.P.-L.); douglasescrivani@gmail.com (D.E.-O.); nataliarruda22@gmail.com (N.A.-C.); bartira@biof.ufrj.br (B.R.-B.); 2 Faculty of Pharmacy, Federal University of Rio de Janeiro, Rio de Janeiro 21941-902, Brazil; jackcvm@hotmail.com (J.C.V.M.); lmcabral2@yahoo.com.br (L.M.C.); 3 Institute of Natural Products Research, Federal University of Rio de Janeiro, Rio de Janeiro 21941-902, Brazil; alcides@nppn.ufrj.br; 4 Department of Chemistry, Durham University, Lower Mountjoy, South Rd, Durham DH1 3LE, UK; p.g.steel@durham.ac.uk; 5 Nanotechnology Engineering Program, Alberto Luiz Coimbra Institute for Graduate Studies, and Research in Engineering—COPPE, Federal University of Rio de Janeiro, Rio de Janeiro 21941-972, Brazil

**Keywords:** chalcone, nanocrystals, milling, leishmaniasis

## Abstract

**Background/Objectives:** Cutaneous leishmaniasis (CL) is a vector-borne neglected disease that can cause permanent deformities. Current chemotherapy based on injections with toxic drugs or oral miltefosine poses many drawbacks, urging the need for new oral therapies. Here, we proposed to increase the bioavailability of NAT22, an intralesionally but not orally active antileishmanial chalcone, through nanocrystallization to promote its oral use in CL. **Methods/Results**: NAT22 nanocrystals were produced using a solvent-free green process of dry and wet milling that reduced NAT22 crystal sizes by around 1500-fold to 257 nm (nanoNAT22). Such reduction in size increased water solubility by 15-fold to 4.3 µg/mL and ensured stability in the absence of stabilizers for at least one month. Of note, nanoNAT22 in aqueous medium was more selective for parasites (SI = 35.2) than NAT22 in 1% DMSO (SI = 7.6). *Leishmania amazonensis*-infected mice treated with oral nanoNAT22 had lesion sizes and parasite loads similar to those achieved with intralesional Glucantime^®^, and significantly smaller than NAT22. **Conclusions**: Together, these results indicate that nanocrystallization is an effective process to render NAT22 chalcone also orally active against CL.

## 1. Introduction

**Figure 1:**
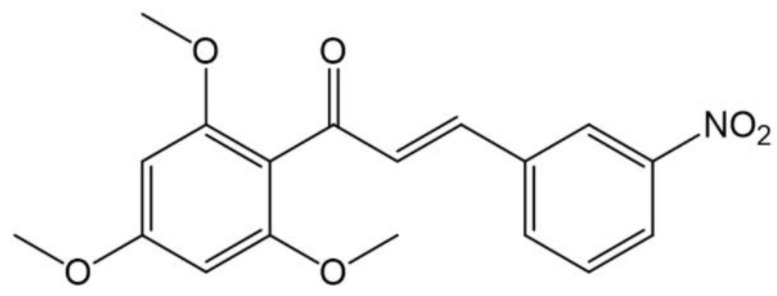
Chemical structure of NAT22 (ChemDraw).

Leishmaniasis is a group of neglected diseases caused by protozoans of the genus Leishmania. It is estimated that around 0.6 to 1 million new cases occur annually worldwide, with cutaneous leishmaniasis (CL) accounting for more than 90% of cases [1]. Although CL is not life-threatening, it can cause disfiguring skin lesions that leave life-long scars and lead to severe social stigma. Pentavalent antimonials such as Glucantime^®^ have been the mainstay of antileishmanial treatment for decades, and amphotericin B formulations and pentamidine have been used in antimonial-resistant cases. However, these drugs are given in long courses of parenteral injections that produce serious side effects and have low patient compliance. Intralesional injections have reduced systemic side effects of antimonials but still require repeated injections and trained medical staff for administration. Miltefosine is the only available oral drug, but it causes gastrointestinal toxicity and is contraindicated for women of childbearing age due to its teratogenicity [2]. Consequently, there is an urgent need for new oral treatments to simplify the treatment of CL [3]. Chalcones are natural products from the flavonoid family with widely reported antileishmanial activity [4,5,6,7]. Previously, we identified a chalcone extracted from *Piper aduncum* inflorescences with strong and selective activity against *Leishmania amazonensis* [8], which served as the basis for the synthesis of more active analogues like 3-nitro-2-hidroxi-4′,6′-dimetoxychalcone (CH8) [9]. More recently, the 3-nitro-2′,4′,6′-trimethoxychalcone (NAT22, Figure 1) was synthesized with a higher yield (89%) than CH8 (18%), and a greater parasite selectivity index compared to CH8 (1489 and 317, respectively). It demonstrated improved intralesional efficacy against CL [10] and strong binding to cytosolic tryparedoxin peroxidase (cTXNPx), a critical parasite target [11]. To enhance patient adherence to treatment, the oral route is recommended. Therefore, we propose the administration of NAT2 chalcone via the oral route. However, NAT22’s low water solubility and large crystal structures are drawbacks for its oral use.

A feasible strategy to improve drug solubility is nanocrystallization, where nanoparticles with drug alone, no lipid, or polymeric carriers have a particle size below 1 µm. Nanocrystals offer several advantages, including increased dissolution velocity, improved adhesion to biological membranes, and enhanced absorption from the gastrointestinal tract [12,13]. Furthermore, the production of nanocrystals ensures enhanced stability in suspensions [14]. Basically, nanocrystals can be produced by three methods: top down, bottom up, or a combination of both [14]. The top-down methods of wet milling and high-pressure homogenization are normally used in products approved by the Food and Drug Administration (FDA) [15,16].

Considering the favorable crystalline nature of NAT22 and its strong therapeutic potential in leishmaniasis, this work aimed to produce NAT22 nanocrystals using a green process of dry and wet milling without the use of organic solvents to promote its oral effectiveness in leishmaniasis.

## 2. Materials and Methods

### 2.1. Materials

NAT22 chalcone was synthesized by Claisen–Schmidt condensation with 99% purity as previously described [10,17]. Analyses of the molecule are presented in the Supplementary Information. Polyvinylpyrrolidone (PVP) K30 was obtained from Labsynth (Diadema, Brazil). Polysorbate 80 was purchased from Biograde (Durham, NC, USA). Dimethyl sulfoxide (DMSO) was obtained from Sigma-Aldrich Co. (St. Louis, MO, USA). High-Performance Liquid Chromatography (HPLC)-grade solvents (acetonitrile and phosphoric acid) were purchased from Tedia (Fairfield, OH, USA). M199 and RPMI medium and heat-inactivated fetal calf serum (HIFCS) were purchased from Cultilab (Campinas, Brazil). Glucantime^®^ (meglumine antimoniate) was obtained from Sanofi Aventis (Lyon, France). A lactate dehydrogenase (LDH) kit was purchased from Abcam (Cambridge, UK) and a Panotipcal fast staining kit was obtained from Laborclin (Pinhais, Paraná, Brazil).

### 2.2. Preparation of nanoNAT22 Nanocrystals

NAT22 nanocrystals (nanoNAT22) were obtained by dry milling followed by wet milling of NAT22. For dry milling, a dry ball mill (Netzsch CGS 10, Selb, Germany) equipped with a 165 mL stainless steel cup and a 30 mm diameter stainless steel unit ball was used. NAT22 (2.5 g) was added to the cup and submitted to a 15 min milling cycle in dry conditions. The first step crystals—NAT22 intermediate crystals (interNAT22)—were recovered (around 80%) by scraping and then submitted to wet milling using a bench top batch mill (Netzsch PE 075, Selb, Germany) equipped with a 600 mL zirconium ceramic cup containing 0.9–1.1 mm diameter zirconium oxide beads (half milling chamber volume) stabilized with cerium oxide (CeraBeads^®^ 1.0, Netzsch, Selb, Germany), as previously described [18]. Briefly, the obtained dry crystals (0.2% *w*/*v*) were dispersed in 280 mL of Milli-Q water containing 0.1% (*w*/*v*) PVPK-30 and 0.6% (*p*/*v*) of polysorbate 80, added to the milling cup with the beads, and stirred at 1700 rpm at 2 °C for 3 h. Then, the whole cup content was sieved through a #40 mesh (0.43 mm), and the sieve and beads were thoroughly washed with distilled water. The filtrate (nanocrystals) was ultracentrifuged (10,000 rpm/1 h) in Thermo Sorvall type WX Ultra (Thermo Fisher Scientific, Waltham, MA, USA) three times with distilled water to remove the surfactants. The sediment was resuspended in 5 mL of distilled water and dried in a glass petri dish at 50 °C/15 h. The obtained yellow nanocrystals were named nanoNAT22. NanoNAT22 and NAT22 were stored at room temperature in a light-protected desiccator jar until use.

### 2.3. Particle Size and Zeta Potential Analysis

NAT22, interNAT22 and nanoNAT22 were resuspended in distilled H_2_O (0.01% *w*/*v*) and dispersed by ultrasonication (UP100H, Hielscher Ultrasonics, Teltow, Germany) for 1 h. While the mean size and polydispersity index (PDI) of NAT22 was determined by laser diffraction using a Mastersizer 2000 (Malvern Panalytical, Malvern, Worcestershire, UK) [10], the interNAT22 and nanoNAT22 dispersions were diluted 1:5 in propylene glycol (5% *v*/*v*) and analyzed by dynamic light scattering (DLS) using a Nano Zetasizer S90 (Malvern Panalytical, Malvern, Worcestershire, UK) [19]. For zeta potential analysis, nanocrystals were resuspended in distilled water (0.1% *w*/*v*) and analyzed using a Zetasizer Advance Lab Blue ZSU3100 (Malvern Panalytical, Malvern, Worcestershire, UK) equipment [20].

### 2.4. Scanning Electron Microscope

Crystal morphology was imaged by scanning electron microscopy (SEM, JSM-6460/LV, JEOL, Tokyo, Japan) [20]. For that, NAT22 and nanoNAT22 powders were dispersed over double face carbon ribbons and coated with gold.

### 2.5. X-Ray Diffraction Analyses

Their crystallinity was confirmed by X-ray diffraction using an XRD-6100 diffractometer (Shimadzu, Kyoto, Japan) set to 40 kV of power, 30 mA of current, using CuKα radiation as an X-ray source, at 1.5418 Å wavelength, and diffraction angle scanning in a 2θ range of 2–80° [18].

### 2.6. Fourier-Transform Infrared Spectroscopy

To evaluate the occurrence of changes in chemical bonds by milling, NAT22, interNAT22 and nanoNAT22 were mixed with KBr and analyzed by Fourier-transform infrared spectroscopy (FTIR) using IR PRESTIGE-21 (Shimadzu, Kyoto, Japan) in the region between 4000 and 400 cm^−1^ [18].

### 2.7. Water Solubility

Solubility was performed using the Shake Flask method [12]. Supersaturated solutions of NAT22 and nanoNAT22 in distilled water were ultrasonicated for 10 min using a Hielscher UP100H sonicator operating at 50% amplitude. After magnetic stirring at 200 rpm overnight, approximately 2 mL of the separation was removed and centrifuged at 10,000 rpm for 30 min. The samples were filtered with syringe filter (pore size 0.45 µm, Merck-Millipore, Ireland) and analyzed by HPLC with an ultraviolet detector (UV–HPLC, Prominence, Shimadzu, Kyoto, Japan).

### 2.8. HPLC Analysis

NAT22 analysis was performed on a Shim-pack VP-ODS (250 × 4.6 mm, with particle size of 4.6 μm) reverse phase column in conditions of isocratic mobile phase containing acetonitrile: 0.01% phosphoric acid (80:20 *v*/*v*) with a flow rate of 1 mL/min, detection at a wavelength of 320 nm and retention time of 4.4 min (running time: 7 min), as previously established [20]. A standard calibration curve was built up by using working standard solutions. Calibration graphs were plotted according to the linear regression analysis, with a high correlation coefficient value (R^2^ = 0.9991).

### 2.9. Particle Size Stability

NAT22 (0.01% *w*/*v*) NAT22 and nanoNAT22 were dispersed in distilled water containing azide (2% *w*/*v*), ultrasonicated as above, and protected from light at 26 °C for 30 days. After different time points, samples were analyzed for size by DLS (Malvern (Malvern Instruments, Malvern, Worcestershire, UK)).

### 2.10. Particle Size Dispersibility

NAT22 and nanoNAT22 were suspended at 2 mg/mL in distilled H_2_O and stirred at 200 rpm overnight. After that, their absorbance, D, 274 nm) was analyzed every 5 min for 60 min using a SPECTRAMAX M5 spectrofluorometer (SpectraMax M5, Molecular Devices, San Jose, Silicon Valley, CA, USA) [21]. At the end of the analysis, macroscopic photos of the dispersions were taken.

### 2.11. In Vitro Assays

#### 2.11.1. Cell Culture

*Leishmania amazonensis* (strain WHOM/BR/75/JOSEFA) and *L. amazonensis*—GFP transfected with the green fluorescent protein (GFP) [22] were cultured at M199 medium supplemented with 10% HIFCS. Bone marrow-derived macrophages (BMDMs) were obtained as described in a previous study [23]. Briefly, cells were differentiated in vitro with L929 cell-conditioned media (LCCM) for 7 days. BMDMs were cultured at RPMI 1640 medium.

#### 2.11.2. Antipromastigote Activity

*L. amazonensis* promastigotes (4 × 10^5^/mL) were plated with medium + 5% HIFCS in 96-well culture plates in the presence of different concentrations (0.4; 2; 10; 50 µM) of drugs for 72 h at 26 °C. NAT22 and nanoNAT22 were directly diluted in M199 prior to addition to parasite cultures. Alternatively, NAT22 and Pentamidine isethionate (Sigma-Aldrich) were pre-diluted in 100% DMSO (Sigma-Aldrich) before addition to cultures (1% DMSO final concentration) [10]. In the last four hours of incubation, resazurin (126 ug/mL, Sigma-Aldrich) was added to each well. At the end, the fluorescence was measured in Microplate Spectrofluorometer (SpectraMax M5, Molecular Devices, San Jose, Silicon Valley, CA, USA) at 555 nm of excitation and 585 nm of emission. Results are represented as the half-maximal inhibitory concentration (IC_50_).

#### 2.11.3. Antiamastigote and Macrophage Cytotoxicity Assays

For antiamastigote activity, BMDMs were allowed to adhere to glass coverslips in a 24-well plate at 2 × 10^5^ cells/well for 24 h/37 °C/5% CO_2_ and then infected with *L. amazonensis* promastigotes (1:10 ratio) in M199 medium plus 5% HIFCS, for 4 h. After washing away non-internalized parasites and during 24 h. After this, cells were treated for 48 h with different concentrations (0.4; 2; 10 and 50 µM) of NAT22, nanoNAT22, or Glucantime^®^. NAT22 was pre-diluted in 100% DMSO before addition to cultures (1% DMSO of final concentration). At the end of treatment, coverslips were stained with Fast Panoptic macrophages and parasites were counted under microscope, and the results are expressed as the number of amastigotes/100 macrophages. For cytotoxicity, the supernatants of BMDM treated as above were colorimetrically assayed for LDH according to the manufacturer (Abcam, Cambridge, UK) instructions. The % LDH release was calculated according to Equation (1). The basal release was cells in medium alone, and maximal release wascells added with 2% Triton [10,20]. The results are expressed as the half maximal cytotoxic concentration (CC_50_), the concentration where the LDH release was 50%.

(1) $$\%\;\mathrm{LDH}=\frac{\left(\mathrm{OD}\;\mathrm{test}\;\mathrm{release}-\mathrm{OD}\;\mathrm{basal}\;\mathrm{release}\right)}{\left(\mathrm{OD}\;\mathrm{maximum}\;\mathrm{release}\;-\;\mathrm{OD}\;\mathrm{basal}\;\mathrm{release}\right)}\times 100$$

The selectivity Index (SI) considers how much more selective the treatment is for the parasite than for the cell, calculated according to Equation (2).

(2) $$SI=\frac{CC50}{IC50\;amastigotes}$$

### 2.12. In Vivo Study

#### 2.12.1. Animals

BALB/c mice (females, 8-week-old) were maintained in a controlled temperature (25 °C), filtered air, 12 h light cycle and given filtered water and commercial food ad libitum throughout the experiments. The protocols using mice were approved by the Federal University of Rio de Janeiro Institutional Animal Care and Use Committee with protocol number CAUAP118.

#### 2.12.2. Efficacy Against CL

BALB/c mice were infected in the ear with 2 × 10^6^ *L. amazonensis*-GFP promastigotes. After 8 days of infection, treatment was started with NAT22 and nanoNAT22 dispersed in polyethylene glycol at a dose of 40 mg/kg (800 µg/100 µL), administered orally by intragastric gavage, 5 times a week for 5 weeks. Glucantime^®^ was injected subcutaneously into the infected ear at 1.5 mg/kg (30 µg Sb/10 µL), 1× per week for 5 weeks. Periodically, animals were weighted for determination of body weight gain, and the ear thicknesses measured with a digital caliper (Mitutoyo, São Paulo, Brazil) for determination of lesion sizes that are expressed as the difference in thickness relative to the uninfected ear. Parasite loads were determined one week after treatment withdrawal (day 52) both by fluorimetry and by limiting dilution assay (LDA), as previously described [24,25].

### 2.13. Statistical Analysis

The results were analyzed by Student *t*-test (solubility) and two-way ANOVA (anti-amastigote and in vivo efficacy). Data are expressed as means ± standard deviation (SD) for in vitro assays and means ± standard error of the mean (SEM) for in vivo efficacy. Differences were considered significant when *p* < 0.05. IC_50_ and CC_50_ values were obtained through non-linear regression with 95% confidence intervals using GraphPad Prism^®^ 7 and 8 software.

## 3. Results and Discussion

### 3.1. Morphology and Size of NAT22 Nanocrystals

**Figure 2:**
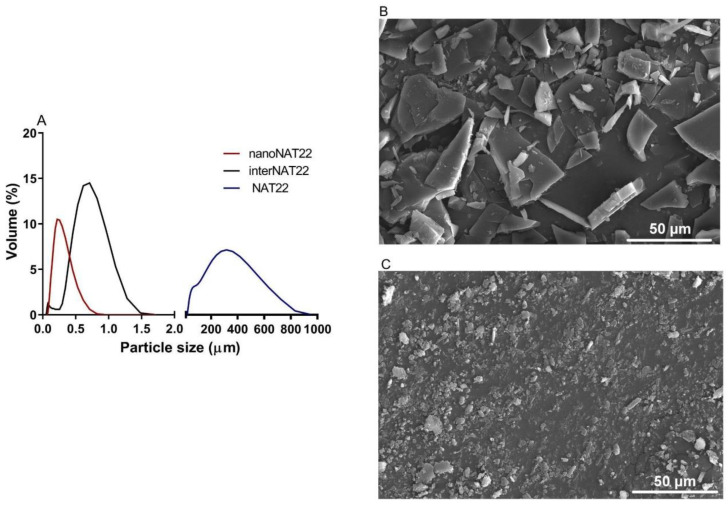
Size distribution and topography of NAT22 crystals. (**A**) Size polydispersion as determined by DLS (interNAT22 and nanoNAT22) and laser diffraction (NAT22). SEM images of (**B**) NAT22; (**C**) nanoNAT22. Size bars are 50 µm.

Chalcones are known for their high crystallinity. Despite their widely described biological activities, the presentation as large crystals when in aqueous solutions hampers their therapeutic use as oral drugs [26]. Chalcone NAT22 has large crystals with a mean diameter of 400 µm and a wide size distribution (d10 = 210 µm; d50 = 309 µm; d90 = 888 µm), with a span = 2.2 (Figure 2A,B and Table 1).

Based on this, the two-step dry milling plus wet milling was approached to obtain nanoNAT22. These are green processes, without the addition of organic solvents. They were carried out on a small scale but are fully industrialized technology [27]. Dry milling normally limits the size reduction to a few micrometers [28]. In dry milling, the intermediate sample interNAT22 was produced with a size around 700 nm and PDI of 0.5 (Figure 2A; Table 1). This result exceeded our expectation, since in the literature, 88 µm crystals were obtained for ibuprofen using this technique [29]. Despite the favorable size and unimodal profile, interNAT22 presented a wide size distribution, with a PDI value > 0.3.

Therefore, it was necessary to perform wet milling to produce nanocrystals in the nanometer size range (200–500 nm) and homogeneous distribution (PDI ≤ 0.3) [15]. In this technique, the size reduction is attributed to mechanical friction and self-friction [30]. NanoNAT22 (dry + wet milling products) had a size of 257 nm and PDI of 0.3 (Figure 2A,C; Table 1), indicating that the milling process was efficient in producing monodisperse nanocrystals, compatible with the unimodal distribution seen in Figure 2A. The process was able to reduce the crystal size by about 1500 times (NAT22 = 400 µm and nanoNAT22 = 257 nm); efficient results in the reduction of nanocrystals were also found for naproxen nanocrystals by wet milling for 1 h, which were reduced by about 500 times [31]. The size of NAT22 was reduced more times, but this depends on the crystal size of the drug. Our wet milling process was conducted in 3 h. According to zeta potential, dry + wet milling made the sample more negatively charged (around 11 times), which increases its stability due to electrostatic repulsion between the particles (Table 1) [12].

### 3.2. Chemical Stability and Crystallinity of NAT22 Chalcone

**Figure 3:**
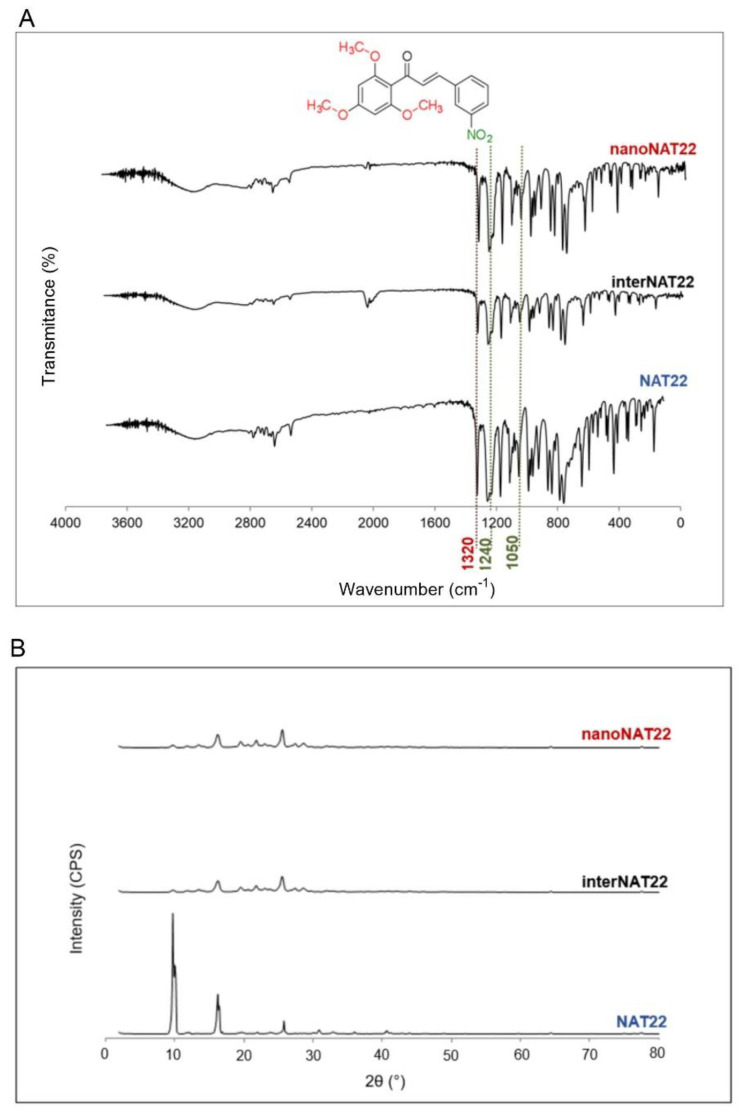
Chemical stability and crystallinity of NAT22 crystals. NAT22, interNAT22, and nanoNAT22 were compared by (**A**) Fourier-transform infrared spectroscopy (FTIR) for chemical stability; and (**B**) X-ray diffraction (XRD) for crystallinity. CPS: counts per second.

The milling process of nanopharmaceuticals should not alter the physicochemical parameters of the molecule [14]. NAT22, interNAT22, and nanoNAT22 were analyzed by FTIR to evaluate possible changes in chemical stability and crystallinity promoted by the milling processes [32]. Figure 3 shows that the three samples (NAT22, interNAT22, and nanoNAT22) presented the same band pattern, regardless of the milling process, indicating that there was no change in the molecule chemical structure (Figure 3A). The main bands of the chalcone groups can be observed in the three samples before and after grinding, with 1250 and 1040 cm^−1^ bands referring to the phenyl alkyl ether group (Figure 3A, red line), and 1600–1530 cm^−1^ and 1390–1300 cm^−1^ referring to nitro vibrations [33]. Another band, corresponding to the C=O axial deformation vibration of the α,β-unsaturated carbonyl, is expected to be observed in the spectrum within the region of 1625 to 1650 cm^−1^ [33]. The absence of this band cannot be attributed to the milling process, as there is no specific band for the α,β-unsaturated carbonyl in the three samples.

In the X-Ray diffractogram shown in Figure 3B, all samples have peaks with similar angles, indicating that the milling did not affect chalcone crystallinity. NAT22 peaks are around 10°, 17°, and 27°, and the same remained after milling [34]. The lower intensity of the peaks may be due to less exposure to X-rays associated with a reduction in crystal size, as seen with other nanocrystals [35,36,37].

### 3.3. Solubility, Dispersibility, and Stability of NAT22 Crystals

**Figure 4:**
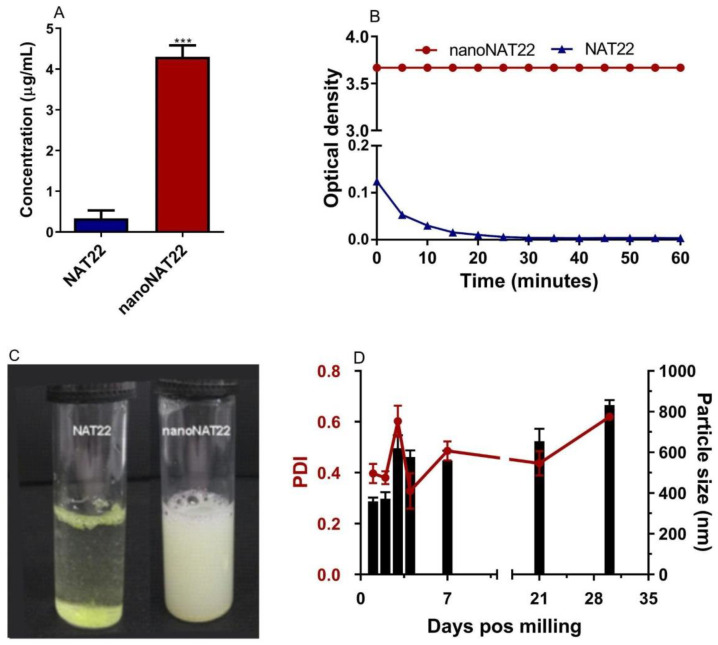
Aqueous solubility, dispersibility, and stability analysis of NAT22 crystals. (**A**) Solubility in water was determined by equilibrium method (shake-flask technique); (**B**) dispersibility was measured by optical density at the indicated times of resting after stirring; (**C**) images of NAT22 (left) and nanoNAT22 (right) suspensions after 60 min of resting; (**D**) variation in size (black) and polydispersion (red) of nanoNAT22 over time. *** *p* < 0.001 in relation to NAT22 in water.

Nanocrystallization is an excellent approach for improving drug solubility due to the larger surface area of the crystals and consequently higher saturation solubility, faster dissolution rate, and increased stability. This concept is explained by the Noyes–Whitney and Ostwald–Freundlich equations [14,16,38]. Nanomilling increased the aqueous solubility of nanoNAT22 (4.4 µg/mL) by 15 times compared to NAT22 (0.3 µg/mL) (Figure 4A). Similar increases in solubility have been observed in other studies involving the milling process, such as those with silybin and diclofenac acid nanocrystals [12,39]. The measured solubility of NAT22 solubility matched the theoretical solubility of 0.253 µg/mL as predicted by small-molecule pharmacokinetic models (pkCSM) [40]. Despite the gain in solubility, nanoNAT22 still did not meet FDA standards (practically insoluble), so crystal dispersibility was also analyzed. NanoNAT22 showed greater dispersion stability when compared to NAT22, remaining with the same OD even after 60 min of analysis, while NAT22 quickly precipitated (Figure 4B,C) [18]. These results show the stability of nanoNAT22 nanocrystals in water as colloidal dispersion [16]. The stability of NAT22 in aqueous suspension was further evaluated by size and PDI over 30 days at room temperature, protected from light. A small increase in crystal size and PDI was observed from day 3 onwards, likely due to the Ostwald Ripening phenomenon, where larger particles grow by aggregating smaller particles [41]. After one month, the size slightly increased to 850 nm, remaining within the nanometric range, even in the absence of stabilizers, unlike diosgenin nanocrystals, which require the combination of pluronic F127 and sodium dodecyl sulfate for one-month stability [36]. Optimizing the formulation of nanocrystals with surfactants might further increase stability. Overall, nanoNAT22 nanocrystals proved to be stable in water, with slight improvement in solubility and significant improvement in dispersion.

### 3.4. Anti-Leishmania Activity of NAT22 Crystals

**Figure 5:**
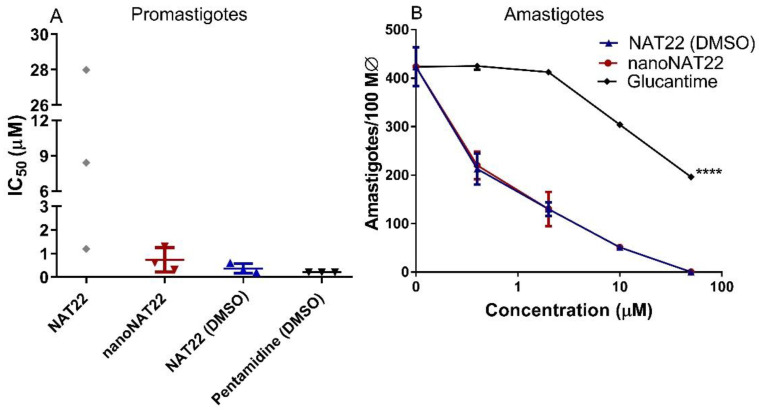
Antileishmanial activity. (**A**) Promastigotes were incubated with different concentrations of drugs for 72h, when parasite viability was fluorometrically determined by resazurin assay. The indicated drugs were directly diluted in aqueous medium in different concentrations, or pre-diluted in DMSO prior to addition to culture maintaining 1% DMSO (NAT22 DMSO and Pentamidine DMSO). Results are expressed as IC_50_ of three independent experiments. (**B**) Intracellular amastigotes were incubated with the indicated drug concentrations for 48h when the cells were stained, counted under microscope, and expressed as amastigotes/100 macrophages. Means ± SD (n = 3), **** *p* < 0.0001 significant in all concentrations in relation to nanoNAT22.

Encouraged by the increased dispersibility and solubility of nanocrystals in water, we compared the antileishmanial activity of NAT22 and nanoNAT22 dispersed in M199 culture medium. The low solubility and dispersibility of NAT22 accounted for the large variation in anti-promastigote IC_50_ values ranging from 1.2 µM to 28 µM in three different experiments (Figure 5A and Table 2). As expected, the nanomilling process improved the anti-promastigote activity, with nanoNAT22 in the M199 medium giving a much more reproducible IC_50_ (0.7 μM). Significantly, this was 19 times higher than NAT22 in the same medium (IC_50_ = 13 μM). Moreover, the IC_50_ of nanoNAT in the M1199 medium was similar to that of fully soluble NAT22 in 1% DMSO (IC_50_ = 0.4 μM). Importantly, similar results of anti-promastigote activity are observed for NAT22 chalcone as a control [11].

As with promastigotes, the large variation in anti-amastigote activity and cytotoxicity when NAT22 was dispersed in medium alone did not allow for the establishment of CC_50_ and SI values. In the intracellular anti-amastigote assay, nanoNAT22 dispersed in the RPMI medium showed the same activity as NAT22 solution in 1% DMSO (Figure 5B), with an IC_50_ equal to 0.6 µM (Table 2), similar to that observed with promastigotes. Despite the higher bioavailability, nanoNAT22 in the RPMI medium was 1.6 times safer (CC_50_ = 12.3 µM) than NAT22 in 1% DMSO (CC_50_ = 7.7 µM); similar results were found by our group for this control [11]. Considering the selectivity index (SI) (SI = CC_50_/IC_50_ amastigote) values, the drug must be at least 10–20 times more selective to the parasite than the cell [42]. With that, nanoNAT22s were 1.5 times more selective for the parasite (SI = 20 for nanoNAT22 in medium and SI = 13 for NAT22 in 1% DMSO) (Table 2). This demonstrates that nanoNAT22 presents greater selectivity and it is in accordance with the defined criteria. We can also highlight the modest increase in the performance of NAT22 chalcone nanocrystals compared to hydroxymethylnitrofurazone nanocrystals prepared by wet milling, as determined by calculating the selectivity index [43].

### 3.5. Oral Efficacy of nanoNAT22 in Murine Model of Cutaneous Leishmaniasis

Nanocrystals are an excellent approach to improve drug solubility/dispersibility, favoring oral administration [28]. The choice of this route is related to greater patient acceptance due to easy administration, no requirement for sterilization, and specialized professional or hospitalization, as happens with some conventional treatments of leishmaniasis, facilitating the treatment of this neglected disease. In addition, the oral route is also in accordance with the guidelines recommended by the Drugs for Neglected Diseases initiative (DNDi) [3].

**Figure 6:**
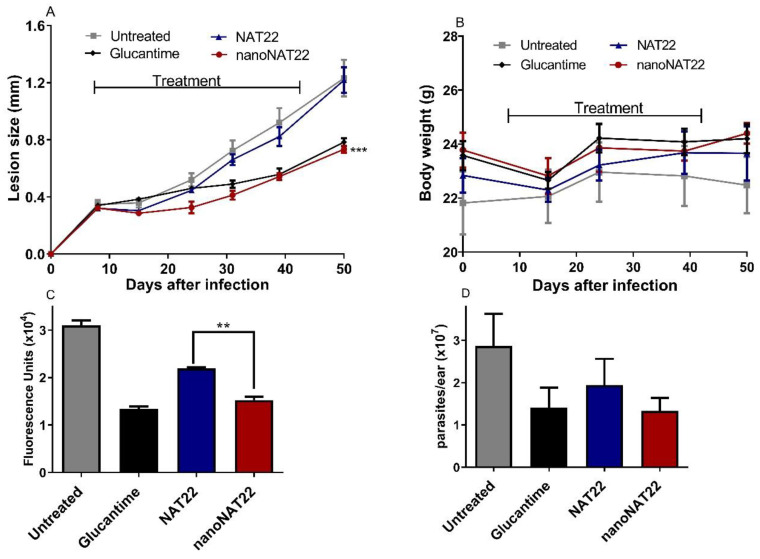
Efficacy of oral treatment with NAT22 nanocrystals in cutaneous leishmaniasis. Mice were infected with *L. amazonensis*-GFP in the ear. On day 8 of infection, they were orally administered with 40 mg/kg of NAT22 or nanoNAT22 in propylene glycol 5×/week, or intralesional injection with Glucantime^®^ (1.5 mg/kg), 1×/week for five weeks. (**A**) Lesion sizes are expressed as the difference of ear thickness in relation to non-infected ears, and (**B**) body weights were measured on the indicated days. Parasite loads in the infected ears were determined on day 52 post infection by (**C**) fluorimetry, and (**D**) limiting dilution assay. Mean ± SEM (n = 5). ** *p* < 0.01; *** *p* < 0.001 in relation to NAT22.

Treatment with nanoNAT22 orally demonstrated greater efficacy than NAT22 in terms of reduction in lesion sizes (*p* < 0.001) and in parasite loads as evaluated by fluorimetry (*p* < 0.01) and LDA that indirectly measures living parasites in the lesion (Figure 6C and Figure 6D, respectively). The effectiveness of nanoNAT22 for oral route was similar to that obtained following treatment with intralesional Glucantime^®^ in terms of reducing lesion size and parasite load (60 and 54%, respectively), and such represents a promising result for a non-invasive route (Figure 6A,C). Importantly, the treatments were not toxic and, despite an initial weight loss, by the end of the treatment all animals gained more weight than the untreated group (Figure 6B). These results demonstrate that nanocrystallization improved the effectiveness of oral chalcone in CL. Similar results have been reported for amphotericin B nanosuspensions in VL, in which a 29% greater reduction in the parasite load was seen, indicating superior oral absorption and efficacy of this formulation [44]. The improvement may be due to the greater adhesiveness of the nanocrystals to the gastrointestinal mucosa, favoring absorption and increasing the residence time in the gastrointestinal tract by increasing the solubility and dissolution of the suspension [38].

## 4. Conclusions

The combined dry and wet milling process was efficient in producing NAT22 nanocrystals (nanoNAT22) with nanometric and homogeneous dispersion without affecting the crystallinity and chemical structure of the molecule. This process subtly improved solubility and substantially increased the dispersibility of NAT22 nanocrystals in aqueous media, keeping the nanocrystals stable and nanosized for at least 30 days in the absence of any stabilizer (size < 1 µm). Nanomilling proved to be an effective method to improve the bioavailability of NAT22 both in vitro and in vivo, promoting good anti-*Leishmania* activity, with a high selectivity index in an aqueous medium and an excellent efficacy of orally administered nanoNAT22. These results demonstrated that nanocrystalline NAT22 is a potent candidate for the oral treatment of cutaneous leishmaniasis.

## Figures and Tables

**Table 1:** Particle size, polydispersity (PDI and span), and surface charge of NAT22 and nanocrystals.

Sample	Particle Size (µM)	PDI	Span	ZP (mV)
NAT22	225 ± 65	NA *	2.2 ± 0.2	−2.3 ± 3.3
interNAT22	0.701 ± 0.05	0.5 ± 0.03	NA *	−7.5 ± 2.1
nanoNAT22	0.257 ± 0.01	0.3 ± 0.04	NA *	−24.6 ± 2.9

Means ± SD (n = 3); * NA: not applicable; ZP: zeta potential.

**Table 2:** In vitro cytotoxicity and parasite selectivity.

Drug	Culture Medium	IC_50_ (µM)		CC_50_ (µM)	SI
		Promastigotes	Amastigotes		
NAT22	Medium	13.0 ± 14.0	ND	ND	ND
nanoNAT22	Medium	0.7 ± 0.5	0.6 ± 0.1	12.3 ± 1	20
NAT22 (DMSO)	1% DMSO	0.4 ± 0.2	0.6 ± 0.1	7.7 ± 0.9	13
Pentamidine (DMSO)	1% DMSO	0.2 ± 0.0	ND	ND	ND
Glucantime^®^	Medium	ND	28.9 ± 0.1	197 *	7

Means ± SD (n = 3); * extrapolated value; IC_50_: half-maximal Inhibitory Concentration. CC_50_: half—maximal cytotoxic concentration. SI (selectivity index): CC_50_/IC_50_ amastigote; ND: not determined.

## Data Availability

Data are available in the article and Supplementary Materials.

## Supplementary Materials

The following supporting information can be downloaded at: https://www.mdpi.com/article/10.3390/pharmaceutics17040399/s1, Supplementary Figure S1. Chromatogram of nanoNAT22 with detection at 320 nm. Supplementary Figure S2. Gas chromatography mass spectrometry (GC-MS). The NAT 22 purity was 99%. Supplementary Figure S3. Mass spectrometry of NAT22. EM (IE) m/z (%) 343 (20), 315 M + (100), 195 (50).

## Abbreviations

The following abbreviations are used in this manuscript:

BMDM	Bone marrow-derived macrophages
CC_50_	Half -maximal cytotoxic concentration
CL	Cutaneous leishmaniasis
DLS	Dynamic light scattering
DMSO	Dimethyl sulfoxide
DNDi	Drugs for Neglected Diseases Initiative
FTIR	Fourier-transform infrared spectroscopy
GFP	Green fluorescent protein
HIFCS	Heat-inactivated fetal calf serum
InterNAT22	NAT22 intermediate crystals
IC50	Half-maximal inhibitory concentration
LDA	Limiting dilution assay
LDH	Lactate dehydrogenase
NA	Not applicable
NanoNAT22	NAT22 nanocrystals
NAT22	3-nitro-2′,4′,6′-trimethoxychalcone
ND	Not determined
OD	Optical density
SD	Standard deviation
PDI	Polydispersity index
pkCSM	Predicting small-molecule pharmacokinetic
PVP	Polyvinylpyrrolidone
SEM	Scanning electron microscopy
SEM	Standard error of the mean
SI	Selectivity index
UV-HPLC	High-Performance Liquid Chromatography with ultraviolet detector
ZP	Zeta potential
