# Supplementary figures and images for: Nanocrystallization Effectively Improves the Oral Efficacy of an Antileishmanial Chalcone
Source: Pharmaceutics. 2025 Mar 21;17(4):399. doi: 10.3390/pharmaceutics17040399 (PMC12030571; doi:10.3390/pharmaceutics17040399)

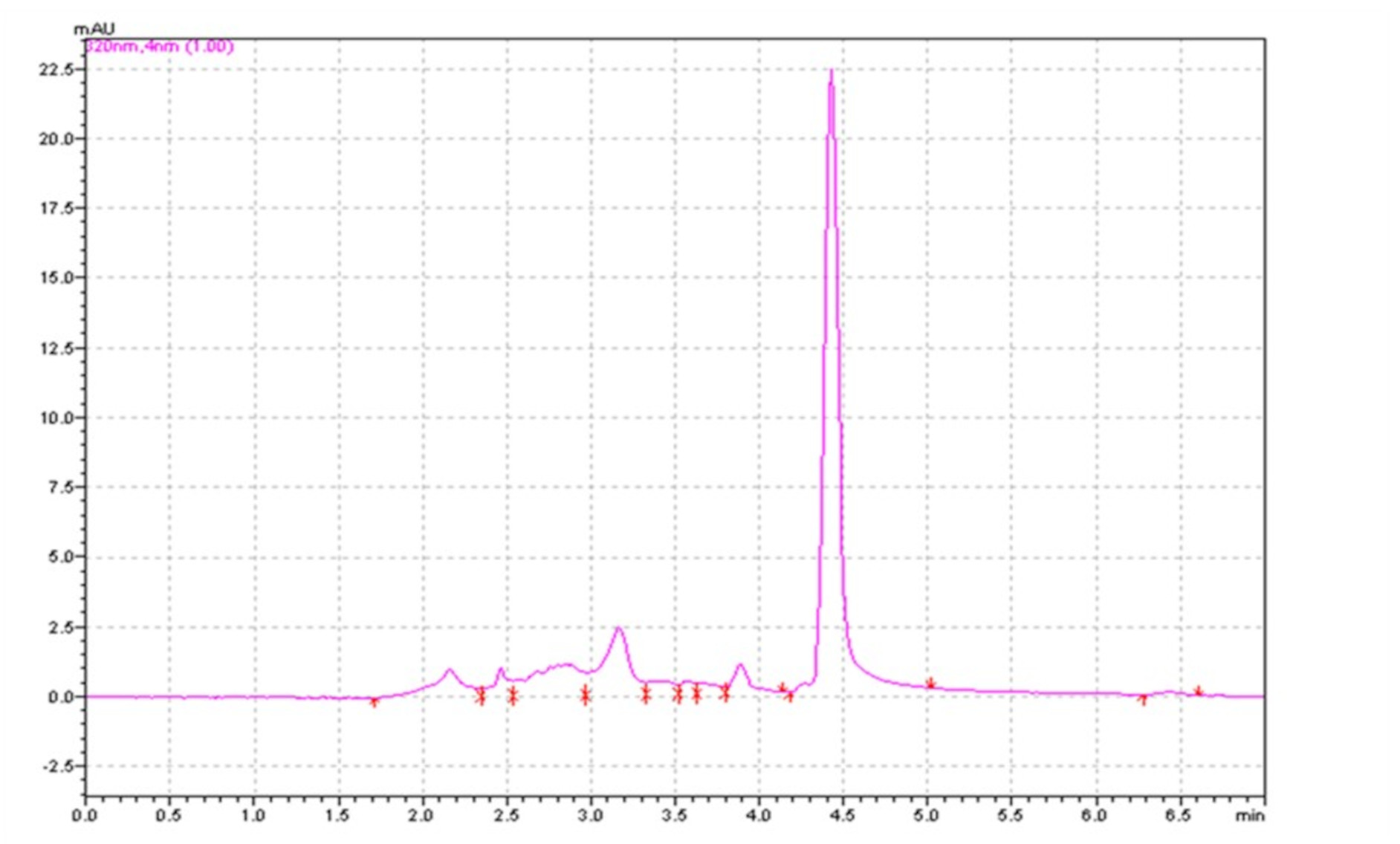

Supplement: Supplementary file 1 [file pharmaceutics-17-00399-s001.zip › Figure S1-Chromatogram.jpg]

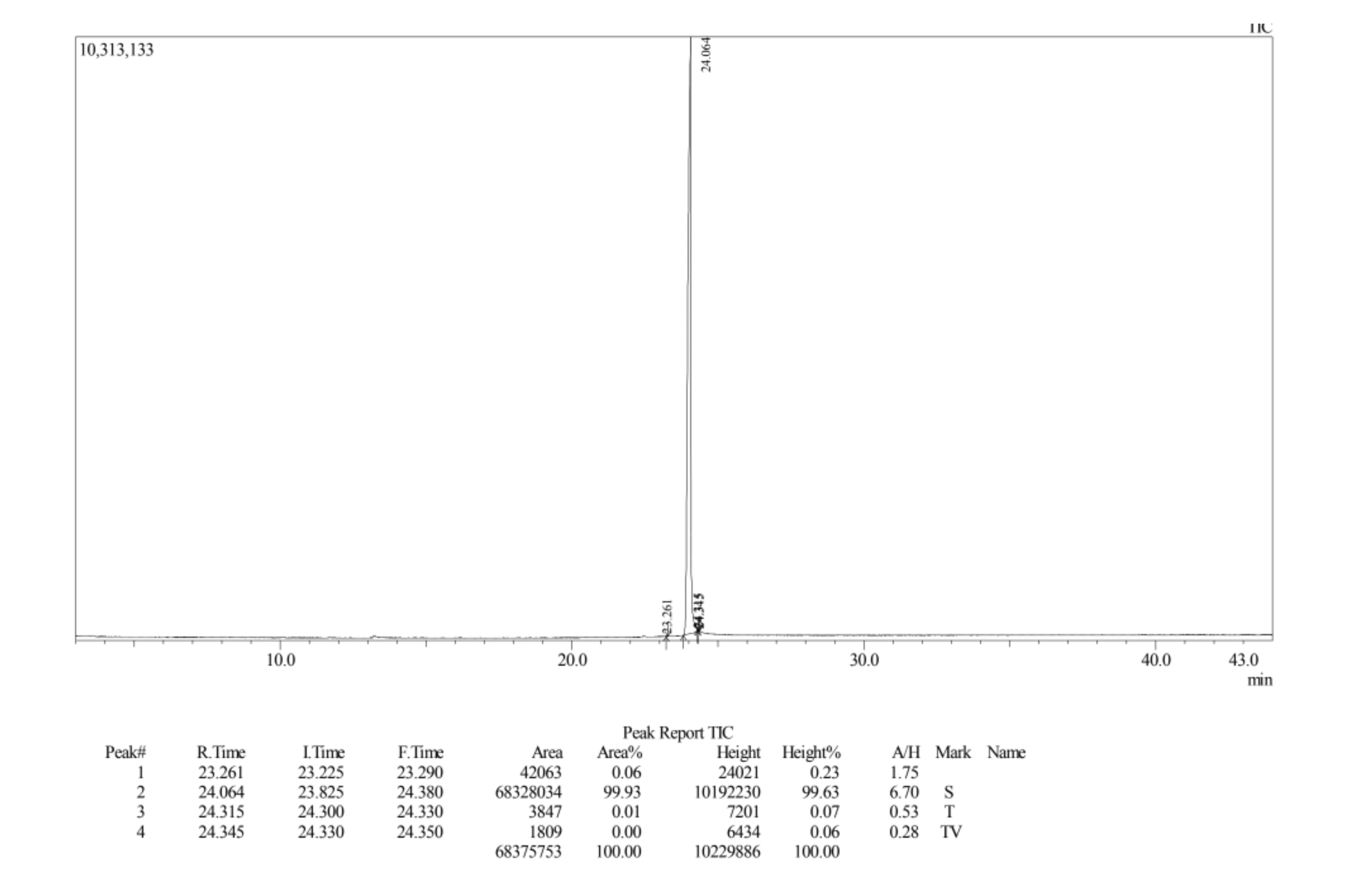

Supplement: Supplementary file 1 [file pharmaceutics-17-00399-s001.zip › Figure S2-CGMS.jpg]

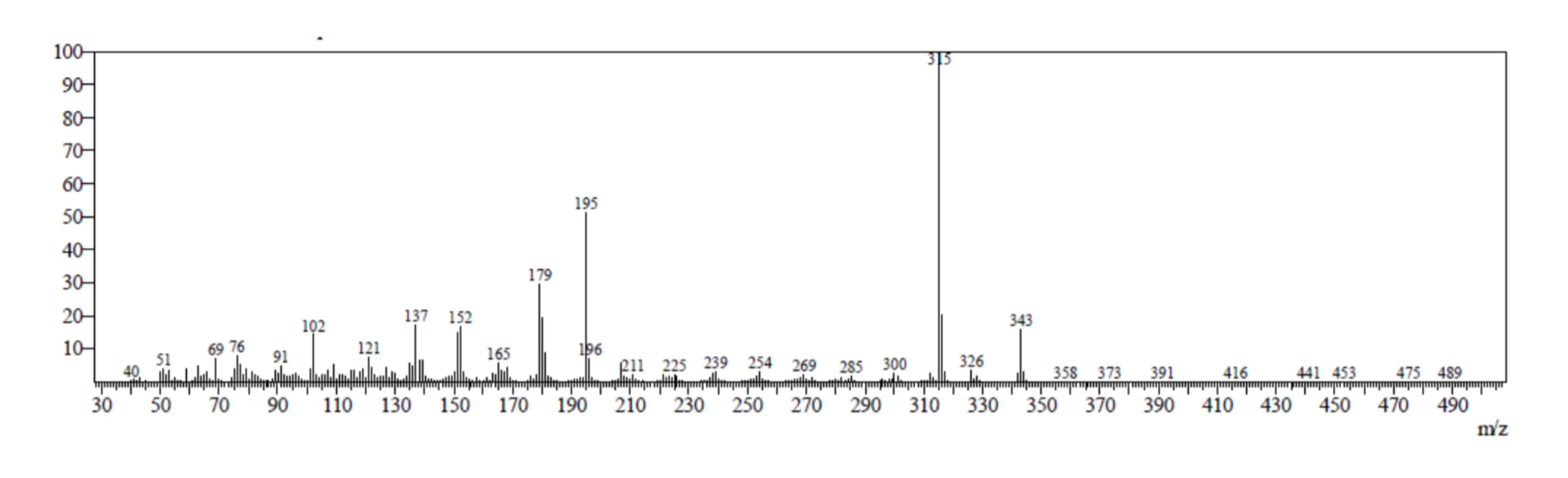

Supplement: Supplementary file 1 [file pharmaceutics-17-00399-s001.zip › Figure S3-mass spectroscopy.jpg]
